# Sleep Apnea in Idiopathic Pulmonary Fibrosis: A Molecular Investigation in an Experimental Model of Fibrosis and Intermittent Hypoxia

**DOI:** 10.3390/life11090973

**Published:** 2021-09-15

**Authors:** Liasmine Haine, Juliette Bravais, Céline-Hivda Yegen, Jean-Francois Bernaudin, Dominique Marchant, Carole Planès, Nicolas Voituron, Emilie Boncoeur

**Affiliations:** 1 UMR INSERM U1272 Hypoxie & Poumon, Université Sorbonne Paris Nord, 93017 Bobigny, France; liasmine.haine@univ-paris13.fr (L.H.); juliette.bravais@outlook.fr (J.B.); celinehivda.yegen@univ-paris13.fr (C.-H.Y.); jf.bernaudin-univ@orange.fr (J.-F.B.); dominique.marchant@univ-paris13.fr (D.M.); carole.planes@aphp.fr (C.P.); nicolas.voituron@univ-paris13.fr (N.V.); 2 Faculté de Médecine, Sorbonne Université, 75012 Paris, France; 3 Service de Physiologie et d’Explorations Fonctionnelles, Hôpital Avicenne, APHP, Hôpitaux de Paris, 93000 Bobigny, France; 4 Département STAPS, Université Sorbonne Paris-Nord, 93000 Bobigny, France

**Keywords:** obstructive sleep apnea, idiopathic pulmonary fibrosis, ER stress, intermittent hypoxia

## Abstract

Background: High prevalence of obstructive sleep apnea (OSA) is reported in incident and prevalent forms of idiopathic pulmonary fibrosis (IPF). We previously reported that Intermittent Hypoxia (IH), the major pathogenic element of OSA, worsens experimental lung fibrosis. Our objective was to investigate the molecular mechanisms involved. Methods: Impact of IH was evaluated on C57BL/6J mice developing lung fibrosis after intratracheal instillation of Bleomycin (BLM). Mice were Pre-exposed 14 days to IH before induction of lung fibrosis or Co-challenged with IH and BLM for 14 days. Weight loss and survival were daily monitored. After experimentations, lungs were sampled for histology, and protein and RNA were extracted. Results: Co-challenge or Pre-exposure of IH and BLM induced weight loss, increased tissue injury and collagen deposition, and pro-fibrotic markers. Major worsening effects of IH exposure on lung fibrosis were observed when mice were Pre-exposed to IH before developing lung fibrosis with a strong increase in sXBP1 and ATF6N ER stress markers. Conclusion: Our results showed that IH exacerbates BLM-induced lung fibrosis more markedly when IH precedes lung fibrosis induction, and that this is associated with an enhancement of ER stress markers.

## 1. Introduction

Idiopathic pulmonary fibrosis (IPF) is the most common form of chronic interstitial lung disease in elderly adults [1,2]. The well-accepted hypothesis to explain the pathogenesis of the disease is related to recurrent micro-aggressions of the alveolar epithelium by various endogenous and exogenous factors (viruses, cigarette smoke, gastroesophageal reflux, pollutants, etc.). Indeed, repetitive alveolar aggressions induce a deregulation of epithelial–mesenchymal interaction and lead to an aberrant repair of the injured epithelium [2]. Alveolar epithelial cell (AEC) phenotype and function are altered and fibroblast proliferation is enhanced. AECs undergo apoptosis [3,4] and/or epithelial–mesenchymal transition (EMT) [5,6]. Moreover, fibroblasts are activated into myofibroblasts. A characteristic micro-environment is formed, principally made up of pro-fibrotic mediators (TGFβ, PDGF, CTGF, etc.) [7]. Accumulation of extracellular matrix (ECM) is then observed, which leads to parenchymal rigidity and contributes to an alteration of lung function [2]. 

Recently, several studies reported the high prevalence of obstructive sleep apnea (OSA) syndrome in IPF patients [8,9]. Indeed, 62% of patients with newly diagnosed IPF present a moderate to severe OSA [8], suggesting that OSA could precede (or at least occur at the same time as) the onset of IPF [8]. Interestingly, the use of OSA therapy by continuous positive airway pressure (CPAP) was shown to be effective on some clinical aspects, improving daily living activities and quality of sleep in IPF patients [10]. However, the impact of OSA on IPF development is unclear [9], and the molecular mechanisms involved in these effects are poorly understood. 

OSA consists of repeated upper airway obstructions leading to transient reduction in inspiratory air flow caused by increased resistance in the upper airways [11]. These obstructions induce hypercapnia and hypoxemia [12]. Thus, OSA is characterized by cyclic episodes of hypoxia–reoxygenation during sleep named “intermittent hypoxia, (IH)” which is particularly deleterious through the generation of oxidative stress and inflammation response and is considered as a key factor in OSA-related comorbidities [13,14]. 

Interestingly, IH exposure, the main pathogenic element of OSA, negatively impacts lipopolysaccharide (LPS)-induced acute lung injury [15]. Indeed, we and others showed that IH worsens the severity of lung fibrosis in rodent models of Bleomycin (BLM)-induced lung fibrosis [16,17] by an undefined molecular mechanism. 

Among the molecular mechanisms that may be involved both in IH and in IPF pathogenesis, endoplasmic reticulum (ER) stress seems to play a significant role. ER stress markers have been found overexpressed in IPF biopsies [18] as well as in mouse models of Bleomycin-induced lung fibrosis [19], and in the heart and lung of mice exposed to intermittent hypoxia [20,21]. ER is involved in calcium homeostasis and in the synthesis, maturation and folding of proteins. When homeostasis is disturbed, following an accumulation of Ca^2+^ or improperly folded proteins, a specific signalling pathway called the Unfolded Protein Response (UPR) is activated, monitoring the ER stress. Three essential branches of the UPR (IRE1–XBP1), (PERK–ATF4) and ATF6 are initiated by the release and the shifting of the GRP78 and GRP75 chaperone proteins on unfolded proteins [22]. As a consequence, UPR will moderate the overall proteins synthesis, promote their degradation, or induce apoptosis or change in cells phenotype by EMT [19,23,24]. Interestingly, ER stress-induced lung apoptosis and fibrosis have recently been observed in the lung of mice exposed to IH [21].

In this study, we document the impact of IH on lung fibrosis severity, and we investigate the molecular mechanisms involved in the worsening effect of IH on lung fibrosis and the impact of a pre-exposure to IH. We show that IH increases ECM deposition and the expression of pro-fibrotic factors. Moreover, we evidence that pre-exposure to IH before the induction of lung fibrosis enhances the expression of ER stress markers. 

## 2. Materials and Methods

### 2.1. Ethical Approval

Experimental protocols, approved by the Charles Darwin Ethics committee, were followed in accordance with European community’s council directive 2010/63/EU for animal care, and French laws for animal care (APAFIS #18309-2019010316127879 v16).

### 2.2. Animals

Experiments were performed in 54 C57BL/6J male mice (Janvier Labs, Le Genest-Saint-Isle, France), about 8 weeks old. All animals were housed in standard conditions in a 12 h/12 h light/dark cycle, at an ambient temperature of 20–22 °C, and had *ad libitum* access to water and food. 

### 2.3. Lung Fibrosis Induction 

Lung fibrosis was induced by a single intra-tracheal instillation of Bleomycin (BLM, Bellon–Sanofi, Aventis, France) at 2 IU/g in 100 µL of PBS. The control group was instilled with 100 µL of PBS. The same qualified experimenter always performed instillations. Weight and survival were monitored daily. Induction and presence of fibrosis injury was confirmed by histological analysis (Hematoxylin–Eosin and Aniline blue staining).

### 2.4. Experimental Design 

To document the impact of the main pathogenic aspect of OSA, i.e., hypoxia–reoxygenation, we exposed mice to intermittent hypoxia. 

**Figure 1:**
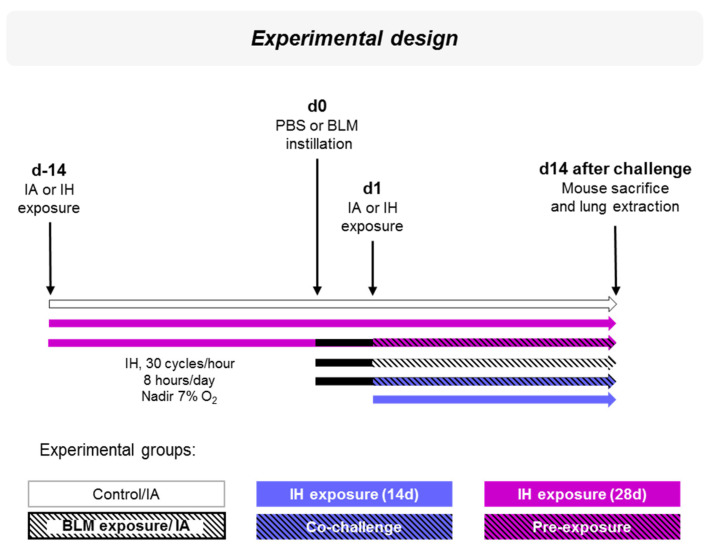
Experimental design of Bleomycin instillation and Intermittent Hypoxia exposure. Mice were exposed to Intermittent Hypoxia (IH; 30 cycles/hour, 8 h/day, Nadir 7% O_2_, magenta color) or intermittent air (IA, white color) (day-14). Fourteen days later, Bleomycin (BLM, black color) or PBS was instilled intratracheally (day 0). Mice were then exposed at d1 to IA or IH; black hatched bars are for BLM/IA exposure, blue color for IH exposure (14 d), blue hatched black for the Co-challenge group, and magenta hatched black for the Pre-exposure. Two weeks later (d14), mice were sacrificed and lungs were extracted for molecular and histological analyses.

Mice were exposed to intermittent hypoxia (IH; 30 cycles/hour, 8 h/day, Nadir 7% O_2_) or intermittent air (IA) (day-14). Fourteen days later, Bleomycin (BLM) (2 IU/g) or PBS instillations were performed (day 0). Mice were then exposed to IH or IA at d1 and sacrificed 2 weeks later (d15). Six experimental groups of at least 5 mice were compared: Control (12 mice); IH exposure (14 d, 9 mice); IH exposure (28 d, 8 mice); BLM exposure (11 mice); Co-challenge (5 mice) and Pre-exposure (9 mice) (Figure 1). 

### 2.5. Intermittent Hypoxia Exposure 

As previously described [17], mice were placed in a customized plexiglas chamber (VelO_2_X, Baker Ruskinn-Alliance Bio Expertise, France) associated with an automated nitrogen/air delivery system (Iconic, Baker Ruskinn-Alliance Bio Expertise, France). Oxygen level was permanently monitored and IH was achieved by adding nitrogen or oxygen in the chamber. The fraction of inspired oxygen (FiO_2_) varies from 21% to 7%. In order to be as close as possible to the kinetics observed in OSA [25], the nadir was reached by adding nitrogen slowly (around 58 s) and the reoxygenation was rapid through oxygen flushing (around 26 s). Mice were exposed for 30 cycles/hour, 8 h/day (during their sleep period) for 14 or 28 days. Control animals were exposed to IA in an identical chamber flushed with the same alternating period with air. 

### 2.6. Lung Extraction

Mice were deeply anesthetized with intra-peritoneal ketamine/xylazine injection (100 mg/kg and 20 mg/kg, respectively) and were sacrificed by section of the abdominal aorta. In order to access the lung, trachea was canulated and a thoracotomy was performed. Lung was rinsed by an injection of physiological saline through the pulmonary artery. Right pulmonary lobes (superior, middle, inferior and post-caval lobes) were isolated through a ligation to the hilium to prevent the passage of the instilled products. The left lobe was inflated and fixed with 4% paraformaldehyde at a pressure of 20 cm H_2_O through the cannula. Heart and lungs were removed *en bloc*. The right lung lobes were separated, frozen in liquid nitrogen and stored at −80°C for RNA and proteins assays. The left lobes were placed in 20 mL 4% paraformaldehyde for 24 h and paraffin embedded. Sections were cut at 5-µm thickness for histology.

### 2.7. Histological Staining

Left pulmonary lobe sections were deparaffinized with xylene, rehydrated and rinsed before Haematoxylin–Eosin (H&E) or Aniline blue (AB) staining. For H&E staining, 5-µm lung sections were consequently stained with Haematoxylin (RAL Diagnostics, Bordeaux, France, 362850-2500, 10 min), 80% ethanol (5 min) and alcoholic Eosin (VWR International, Rosny-sous-Bois, France, 10047103, 2 min). For AB staining, 5-µm lung sections were stained with aniline blue solution (VWR International, Rosny-sous-Bois 34015.182, 5 min), and rinsed with 0.1% acetic acid solution (2 min). After H&E or AB staining process, sections were washed, mounted, dehydrated with ethanol, and cleared with xylene and cover-slipped using mounting medium for microscopic analysis.

### 2.8. Lung Injury and Fibrosis Quantification

Lung injury was estimated by the quantification of tissue density after H&E staining using HistoLab® Image Analysis Software version 10.1 (Microvision Instrument, Every, France). The normally aerated alveolar spaces area was calculated as well as the total area of the examined left lung section. Subsequently, the area occupied by the injured tissue was deduced and reported as the percentage of the total lung section. 

Fibrosis (i.e., collagen deposition) was evaluated by the quantification of the deep blue AB staining using HistoLab^®^ Image Analysis Software version 10.1 (Microvision Instrument, Every, France). Collagen deposition area was reported to the total area of the left lung section.

### 2.9. Quantitative Real-Time Reverse Transcription–Polymerase Chain Reaction

Total RNA was isolated from right lung tissues (superior lobe) using Trizol reagent protocol (Thermo Scientific, Illkirch, France). In this process, 1 µg of total RNA was used to synthesize cDNA using Maxima first strand cDNA synthesis kit for RT-qPCR (Thermo Scientific, Illkirch, France) according to the manufacturer’s instructions. 

Resulting cDNAs were diluted to 1/10^e^ and amplified by quantitative real-time polymerase chain reaction (qPCR) using SYBR Green (Absolute SYBR Green Rox Mix; Thermo Scientific, Illkirch, France) with specific gene primers designed to have 25–30 cycle threshold values (Table 1). 

As an internal control, 18s RNA was used to verify the equality of cDNA quantity in each condition. Relative quantification of gene expression was performed using the 2^−ΔΔCt^ method reported to basal condition.

### 2.10. Lung Protein Extraction and Immunoblotting Analysis

Total proteins were isolated from right lung (inferior lobe). Tissues samples were homogenized on ice using an Ultra-Turrax homogenizer in RIPA protein extraction buffer (50 mM TRIS-HCl, 150 mM NaCl, 1% NP40, 0.1% SDS, 0.5% sodium deoxycholate with adjusted pH 3.7) supplemented with Complete Protease Inhibitor Cocktail (Thermo Scientific, Illkirch, France). Proteins concentration was determined using BCA kit (Thermo Scientific, Illkirch, France) according to the manufacturer’s instructions. 

Forty micrograms (40 µg) of proteins were used for western blotting according to standard procedures. Briefly, proteins were electrophoresed in denaturized conditions (SDS-PAGE), transferred to a nitrocellulose membrane and blocked 1 h at room temperature in 0.1% TBS-Tween solution supplemented with 5% fat-free milk. Membranes were then blotted at 4 °C overnight with specific primary antibodies (Table 2). Protein ratios or expression levels were normalized to β-actin protein expression level, used as an equal loading control.

### 2.11. Data and Statistical Analysis

Statistical analyses were performed using GraphPad Prism^®^ software version 9. Results are presented as means ± standard deviations (SD). D’Agostino–Pearson omnibus normality test was used to assess the distribution of the data. 

Friedman test followed by Dunn’s multiple comparisons test was performed to estimate the difference in mice weight as compared to initial weight. T-test or Mann–Whitney test were applied to compare the difference in mice weight between control group and other three groups and between Co-challenge or Pre-exposure condition versus BLM-challenged group. Differences were considered significant when *p* < 0.05.

Mann–Whitney test was performed to compare the effect of Bleomycin challenge, intermittent hypoxia exposure (14 d or 28 d), Co-challenge or Pre-exposure condition versus control group at the histological and molecular levels and the effect. This test was also applied to compare differences between the Co-challenge group or Pre-exposure group versus BLM-exposure group. Differences were considered significant when *p* < 0.05.

## 3. Results

### 3.1. Intermittent Hypoxia Promotes Tissue Remodelling and Collagen Deposition That Worsens BLM-Induced Lung Fibrosis

Animals were exposed to IH and/or BLM as presented in Figure 1. 

**Figure 2:**
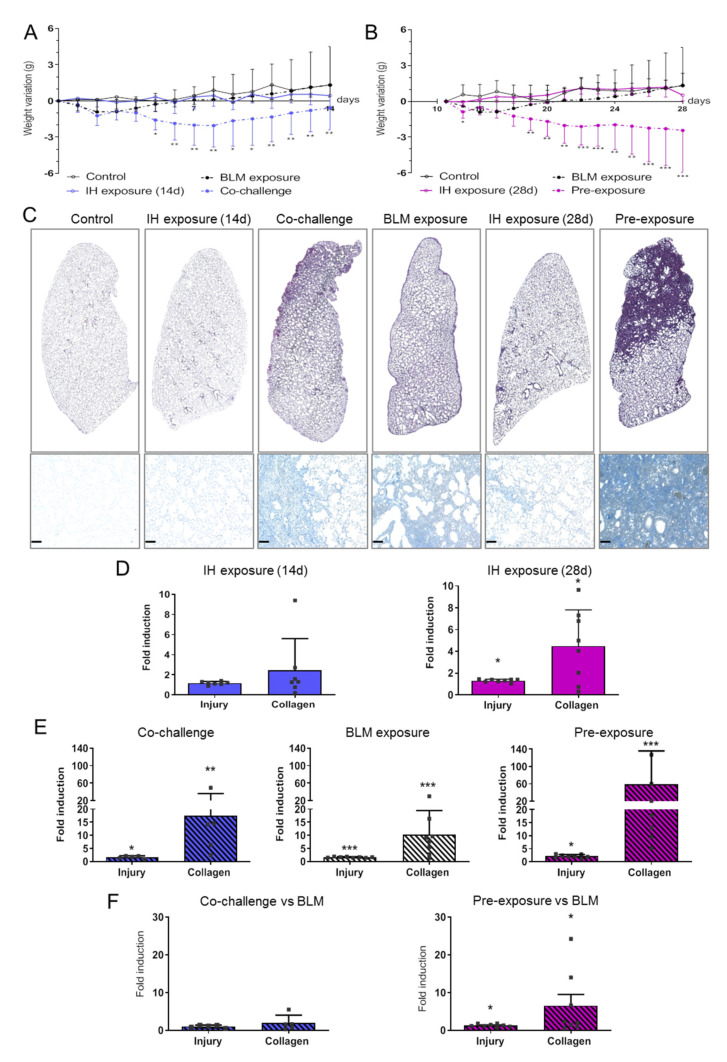
The effect of Bleomycin instillation and or intermittent hypoxia exposure on lung injury and lung collagen deposition. Bleomycin instillation and intermittent hypoxia exposure were performed according to the experimental design. (**A**) Variation of mouse body weights in the Co-challenge (14 d) protocol; (**B**) Variation of mouse body weights in the Pre-exposure (28 d) protocol. Weight loss or gain was calculated by the difference between the daily mice weight (dx) and the initial mice weight at day 0 (Δ weight = dx − d0). Friedman test was performed to evaluate the difference in delta weight relative to initial weight (Δ weight (dx − d0) versus initial weight at d0). Difference on delta weight between Co-challenged or Pre-exposed group versus BLM exposure group was evaluated by T-test or Mann–Whitney test (* *p* < 0.05, ** *p* <0.01, *** *p* < 0.001). (**C**) Histological staining of lung sections. Haematoxylin-eosin (H&E) staining was used to assess lung remodelling (upper panel) and Aniline Blue staining (AB) for visualization of lung collagen deposition (lower panel). Five µm sections of paraffin-embedded left lung were prepared from mice exposed to the experimental design. H&E results were represented by cartography of total left lung section area using Cartography^®^ software. Pictures shown are representative of multiple experiments (n ≥ 5) (**D**,**E**) Lung injury was estimated by the quantification of tissue density after H&E staining using HistoLab^®^ Image Analysis Software. The normally aerated alveolar spaces area was calculated as the total area of the examined left lung section. Subsequently, the area occupied by the injured tissue was deduced and reported as the percentage of the total lung section. Fibrosis *ie.* collagen deposition was evaluated by the quantification of the deep blue AB staining using HistoLab^®^ Image Analysis Software. Collagen deposition area was reported to the total area of the left lung section. The effects of IH exposure (14 d, n = 7 or 28 d, n = 8), BLM exposure (n = 8), Co-challenge (n = 5) or Pre-exposure (n = 8) were presented as fold induction normalized to the mean value of control group and reported to 1. Each “■” represent one animal included in the protocol. Raw data were submitted to Mann–Whitney test to compare each group with control group (Control group versus others groups, * *p* < 0.05, ** *p* < 0.01, *** *p* < 0.001). Y axis is represented in two segments with both bottom and top at 50% length. (**F**) Co-challenge and Pre-exposure experimental conditions were compared to BLM exposure alone. The effects of Co-challenge (n = 5) or Pre-exposure (n = 8) are presented as fold induction normalized to the mean value of BLM exposure group and reported to 1. Each “■” represent one animal included in the protocol. Raw data were submitted to Mann–Whitney test to compare each group with BLM exposure group (BLM exposure group versus others groups, * *p* < 0.05).

No mortality was observed in the six groups: control, IH exposure (14 d), Co-challenge, BLM exposure, IH exposure (28 d), Pre-exposure (data not shown). Intermittent hypoxia exposure or BLM instillation alone did not induce a body weight loss after 14 or 28 days (Figure 2A and Figure 2B, respectively). As compared to BLM alone, double challenge of BLM instillation and IH exposure leads to a significant weight loss from the fifth days after BLM instillation (Figure 2A, *p* = 0.01). The pre-conditioning with 14 days of IH before the double challenge (Pre-exposure group) leads to a significant weight decrease occurring the next days after BLM instillation (Figure 2B, *p* = 0.032). Impact of IH alone or in co-treatment with BLM on lung fibrosis was documented by the measurement of lung injury and collagen deposition on tissue sections (Figure 2C). Lung injury was documented by Haematoxylin and Eosin (H&E) staining (Figure 2C upper panel), and fibrotic areas were estimated as the deep blue areas after aniline blue (AB) staining (Figure 2C lower panel). 

A 14-day IH exposure did not induce remarkable modification in lung morphology, and no significant increase in lung injury and collagen deposition has been observed (Figure 2C,D). Co-challenge and Pre-exposure with IH as well as BLM exposure alone induced architectural changes with epithelium damage and fibrosis evidenced by collagen deposition (Figure 2C). Double challenge induced parenchymal remodelling with compact lung structure and alveolar collapse (H&E staining, Figure 2C), a reduction of alveolar spaces (Figure 2C), and an excessive extracellular matrix synthesis and collagen deposition (Figure 2C,D). This observation was more pronounced when mice where pre-exposed 14 days to IH before the double challenge. In this condition, we observed a severe interstitial thickening and an accentuated alveolar collapse with a total loss of alveolar space (H&E staining, Figure 2C upper panel). Furthermore, IH pre-exposure induced an excessive extracellular matrix synthesis with an important proportion of collagen deposition (AB staining, Figure 2C lower panel). These observations were confirmed by the quantification of histological abnormalities, showing a significant increase in tissue remodelling and collagen deposition (Figure 2D,E). When comparing the Co-challenge or the Pre-exposure effect to the BLM impact on lung injury, we observed that only the Pre-exposure worsens the injury and collagen deposition observed in the BLM exposure alone (Figure 2F). 

### 3.2. Intermittent Hypoxia Increases Collagen and the Production of Pro-Fibrotic Mediators and Contributes to its Worsening Effect on BLM-Induced Lung Fibrosis

**Figure 3:**
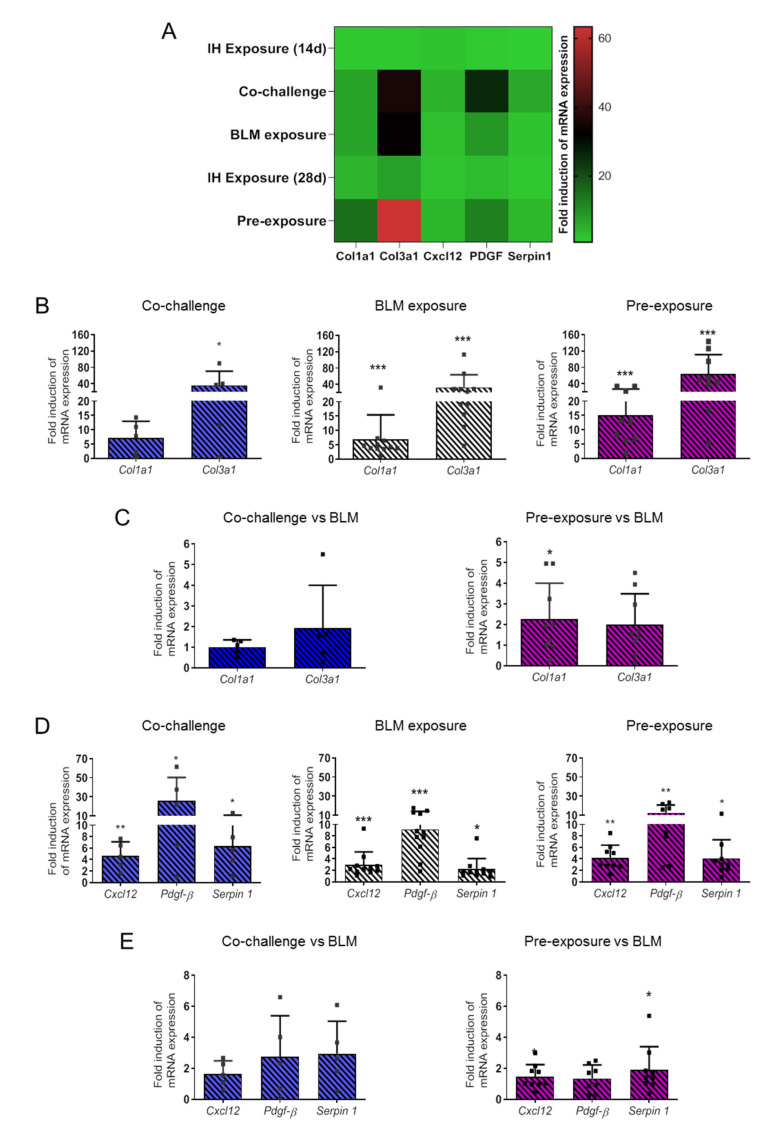
The effect of Bleomycin instillation and/or intermittent hypoxia exposure on collagen and pro-fibrotic marker expression. Bleomycin instillation and intermittent hypoxia exposure were performed according to the experimental design. (**A**) Heatmap representing mRNA expression of Collagen 1a1 (Col1a1), Collagen 3a1 (Col3a1) and pro-fibrotic markers (Cxcl12, Pdgf-β and Serpin1) quantified by RT-qPCR. RT-qPCR were performed on right lung total lysates (superior lobe) from mice exposed to each experimental protocol. Relative quantification of mRNA gene expression was performed using the 2^−ΔΔCt^ method with 18S as an internal control. Effect of each protocol was presented as fold induction normalized to the mean value of control group and reported to 1. (**B**) Statistical representation of mRNA expression of Collagen 1a1 (Col1a1) and Collagen 3a1 (Col3a1) in the Co-challenge, BLM exposure and Pre-exposure group. Each “■” represent one animal included in the protocol. Raw data were submitted to Mann–Whitney test to compare each group with control group (Control group versus others groups, * *p* < 0.05, ** *p* < 0.01, *** *p* < 0.001 with IH exposure (14 d, n = 7 or 28 d, n = 7), BLM exposure (n = 11), Co-challenge (n = 5) or Pre-exposure (n = 9). Y axis is represented in two segments with both bottom and top at 50% length. (**C**) The effects of the double challenge BLM/HI in the Co-challenge or in the Pre-exposure experimental conditions on Collagen 1a1 (Col1a1) and Collagen 3a1 (Col3a1) were compared to BLM exposure alone. The effects of Co-challenge (n = 5) or Pre-exposure (n = 9) are presented as fold induction normalized to the mean value of BLM exposure group and reported to 1. Each “■” represent one animal included in the protocol. Raw data were submitted to Mann–Whitney test to compare each group with BLM exposure group (BLM exposure group versus others groups, * *p* < 0.05). (**D**) Statistical representation of mRNA expression of pro-fibrotic markers (Cxcl12, Pdgf-β and Serpin1) in the Co-challenge, BLM exposure and Pre-exposure group. Each “■” represent one animal included in the protocol. Raw data were submitted to Mann–Whitney test to compare each group with control group (Control group versus others groups, * *p* < 0.05, ** *p* < 0.01, *** *p* < 0.001 with IH exposure (14 d, n = 7 or 28 d, n = 7), BLM exposure (n = 11), Co-challenge n = 5) or Pre-exposure (n = 9). Y axis is represented in two segments with both bottom and top at 50% length. (**E**) The effects of the double challenge BLM/HI in the Co-challenge or in the Pre-exposure experimental conditions on pro-fibrotic markers (Cxcl12, Pdgf-β and Serpin1) were compared to BLM exposure alone. The effects of Co-challenge (n = 5) or Pre-exposure (n = 9) are presented as fold induction normalized to the mean value of BLM exposure group and reported to 1. Each “■” represent one animal included in the protocol. Raw data were submitted to Mann–Whitney test to compare each group with BLM exposure group (BLM exposure group versus others groups, * *p* < 0.05).

Expression of *Collagen 1a1*, *Collagen 3a1*, and pro-fibrosing mediators *Cxcl12*, *Pdgf-β* and *Serpin 1* were quantified by RT-qPCR in each condition. Both 14 d and 28 d IH exposure significantly induced expression of *Col1a1* and *Col3a1* gene expression (data not shown) but the magnitude of this effect was relatively low as compared to other challenges (Figure 3A). Interestingly, as highlighted in the heatmap (Figure 3A), Co-challenge and Pre-exposure induced strong up-regulation of *Col3a1* and *Pdgf-β* as compared to BLM exposure (Figure 3A). BLM exposure, Co-challenge and Pre-exposure induced a significant increase in *Col1a1*, *Col3a1*, *Cxcl12*, *Pdgf-β* and *Serpin 1* gene expression as compared to control condition (Figure 3B and Figure 3C, respectively). The major effect of IH exposure on BLM-induced pro-fibrotic factors is observed in the Pre-exposure group with a strong induction of *Col3a1* expression (red in the heatmap).

When comparing the Co-challenge or the Pre-exposure effects to the BLM alone effect on collagen and pro-fibrotic mediators, we observed that only the Pre-exposure condition emphasizes *Col1a1*, *Cxcl12* and *Serpin 1* expression already observed in the BLM exposure alone (Figure 3C,E). 

### 3.3. Intermittent Hypoxia Modulates ER Stress Response That Enhances BLM Impact on ER Stress Markers

**Figure 4:**
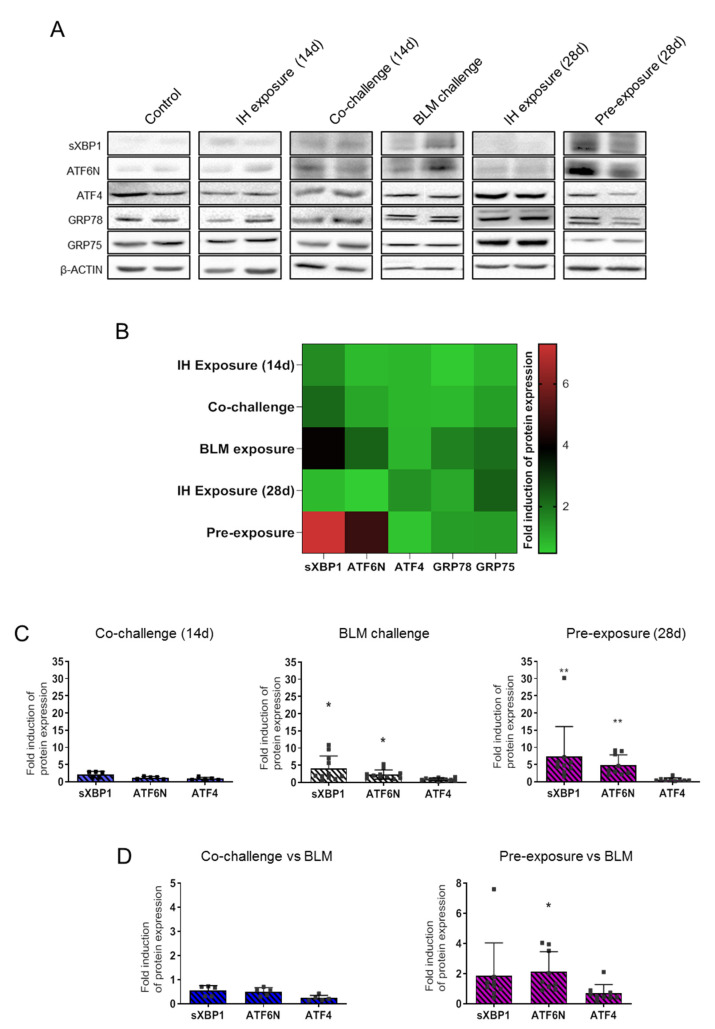
The effect of Bleomycin instillation and/or intermittent hypoxia exposure on ER stress markers and chaperones expression. Bleomycin instillation and intermittent hypoxia exposure were performed according to the experimental design. (**A**) Western blot analysis of ER stress markers sXBP1, ATF6N, ATF4, and chaperones GRP78, GRP75 protein expression. Western blots were performed on right lung total lysates (inferior lobe) from mice exposed to each experimental protocol. Blots shown were representative of four experiences. (**B**) Heatmap representing the quantification of protein expression of ER stress markers (sXBP1, ATF6N, ATF4) and chaperones (GRP78, GRP75) analysed by western blot. Densitometry analysis of each band has been obtained with Image Lab software. Protein expression was normalized to β-actin. Expression of each protein tested is presented as a mean value expressed in fold induction normalized to the mean value of control group reported to 1. (**C**) Statistical representation of the quantification of ER stress markers expression in the Co-challenge, BLM challenge and Pre-exposure group. Each “■” represent one animal included in the protocol. Raw data were submitted to Mann–Whitney test to compare each group with control group (Control group versus others groups, * *p* < 0.05, ** *p* < 0.01) with IH exposure (14 d, n = 9 or 28 d, n = 8), BLM exposure (n = 11), Co-challenge (n = 5) or Pre-exposure (n = 9). (**D**) The double challenge BLM/HI in the Co-challenge or in the Pre-exposure experimental condition were compared to BLM alone exposure. The effects of Co-challenge (n = 5) or Pre-exposure (n = 9) are presented as fold induction normalized to the mean value of BLM exposure group and reported to 1. Each “■” represent one animal included in the protocol. Raw data were submitted to Mann–Whitney test to compare each group with BLM exposure group (BLM exposure group *versus* others groups, * *p* < 0.05).

Protein level expression of the three major transcription factors induced for the resolution of ER stress, i.e. sXBP1, ATF6N and ATF4, and the expression of the mitochondria-to-ER chaperone GRP75 and GRP78 were analysed by western blot in each condition (Figure 4A,B).

Western blots analysis demonstrated that long-time exposure (28 days) of mice to IH induced an increase in the expression of the mitochondria-to-ER chaperone GRP75. Interestingly, whereas BLM exposure alone induced the expression of either the ER chaperone GRP78 and the mitochondria-to-ER chaperone GRP75, no induction was observed either in the Co-challenge or Pre-exposure experimental condition (Figure 4B). Expression of GRP78 was slightly induced in the BLM exposure condition and GRP75 in the BLM condition and in the long time IH exposure (28 d) condition.

Analysis of ER stress markers modulation showed an increase in the expression of sXBP1 in each condition, except for long IH exposure (28 d). ATF6N was strongly induced in the BLM and Pre-exposure condition, while ATF4 was only induced in the long time IH exposure (28 d) (Figure 4B). Statistical analyses of the expression of sXBP1, ATF6N and ATF4 (Figure 4C) confirmed the observations made in Figure 4A,B. BLM exposure significantly induced sXBP1 and AT6N expression, an effect which was not observed in the Co-challenge group. Finally, the major increase in the expression of both sXBP1 and ATF6N in the Pre-exposure group observed in the Heatmap representation (Figure 4B) was confirmed by the statistical analyses in Figure 4C.

When comparing the Co-challenge or the Pre-exposure effects to the BLM alone effect on collagen and pro-fibrotic mediators, we observed that only the Pre-exposure condition emphasizes *ATF6N* expression already observed in the BLM exposure alone (Figure 4D). 

## 4. Discussion

In the present study, we proposed to investigate the link between OSA and IPF, to explore the impact of presenting OSA before IPF, and to decipher the mechanisms involved in the worsening effect of intermittent hypoxia on the severity of lung fibrosis previously observed [17]. Here, we hypothesized that this effect would be secondary, at least in part, to an increased cellular ER stress. 

To address this question, we used a mouse model of lung fibrosis induced by intratracheal instillation of Bleomycin. Different concentrations of Bleomycin are used to induce pulmonary fibrosis depending on the mice strain, the source of the Bleomycin as well as the housing conditions in the animal facility [26]. This widely-characterized model recapitulates at day 14 after BLM instillation many features of human lung fibrosis i.e. collagen deposition within the alveolar interstitium, a thickening of the alveolar walls and masses of fibrosing-type lesions on the lung surface [27]. In our experimental conditions, no significant aggravating effect of IH on lung fibrosis severity was observed by histological analysis in mice Co-challenged with IH and BLM. However, as compared to BLM alone, when mice were pre-exposed to IH, a significant weight loss, and an aggravated lung fibrosis is observed, characterized by a more reshaped lung parenchyma with an excessive increase in collagen deposition.

Twenty eight days of IH exposure alone significantly increased the induction of *Collagen 1a1* and *Collagen 3a1* mRNA expression in whole lung extracts and protein deposition in lung parenchyma, two fundamental events in fibrosis development [28]. Furthermore, and in agreement with the study of Shi et al. [21], IH significantly increased the *Pdgf-β* and *Cxcl 12* mRNA pro-fibrotic markers expression. *Cxcl12* and *Pdgf-β* are well identified in the pathogenesis of IPF [29] and well known to induce the proliferation, and migration of fibroblasts [30], and to chemoattract circulating fibrocytes [31]. 

As reported by others, we found an increase in the expression of extracellular matrix component (collagens) and pro-fibrotic *Cxcl12*, *Pdgf-β* and *Serpin 1* mediators in mice challenged with BLM [32]. Interestingly, as compared to mice treated with BLM alone, we observed an additive effect with increased expression of *Collagen 1a1*, *Cxcl12 and Serpin 1* mRNA expression when mice were pre-exposed to IH before BLM challenge. Interestingly, IH and BLM both induced the expression of *Serpin 1* (PAI-1 in Human). PAI-1 was shown to be up-regulated in AEC II cells from IPF patient [33], and could play a critical role in the development of lung fibrosis [34] through induction of cell senescence [35]. Interestingly, cellular senescence has also been proposed to explain the pathophysiology of IPF [36], and its potential role in the aggravating effect of IH cannot be excluded.

Twenty-eight days IH exposure also induced an increase in the expression of ATF4, and GRP75 mitochondria-to-ER chaperone ER stress markers. These findings are in agreement with a recent study that showed an induction of ATF4 ER stress marker in lung after four weeks of IH exposure [21]. In the latter study, Shi et al. suggested that the lung ER stress induced by IH was mostly mediated by activation of the PERK/ATF4 pathway. The activation of PERK/ATF4 pathway could also amplify an anti-oxidative response via NRF2 transcription factors activation [37]. Moreover oxidative stress response was described as a major deleterious consequence of IH in lung [38]. Indeed, it has been recently proposed that the NF-kB/NRF2 signaling pathway could partly explain the deleterious impact of IH on lung fibrogenesis [39].

Induction of sXBP1, ATF6N and GRP78 ER stress markers was considered as a common molecular feature of IPF, also observed in BLM-induced lung injury in mice [19,32] The origin of ER stress observed in lung biopsies from IPF patients and mice lung in experimental fibrosis is not completely understood and the implication of a hypoxic microenvironment of AEC cells has been proposed [19,40]. IH effects on the induction of ER stress markers were widely studied in several organs. In particular, the induction of GRP78, CHOP, sXBP1 mRNA has been reported in cardiomyocytes of mice in a model of ischemia-reperfusion injury [20]. Intriguingly, IH exposure alone, BLM exposure alone and the double challenge induced distinct UPR pathways. IH exposure activated the ATF4 branch of the UPR, BLM exposure the XBP1/ATF6 branch, and the double challenge exacerbated the activation of ATF6. The precise mechanism of this switch remains to be determined. We could hypothesize that activation of ATF6N directs the cells to a senescent phenotype (as proposed by Kim et al. [41]) mainly observed in IPF [36] and described as a consequence of both exposure to IH [42] and to BLM treatment [43]. Future studies are needed to decipher the implication of cell senescence and its relationship with ER stress in the worsening effect of IH in lung fibrosis, and will be essential to understand the molecular mechanisms involved in lung fibrogenesis. 

Our study aimed to understand the impact of OSA on IPF by exploring the effect of intermittent hypoxia—the key pathogenic element of OSA—on the severity of experimental lung fibrosis. This approach has some limitations. For instance, the hypercapnia component present in OSA (due to the physical obstruction of the throat) was not integrated in our model, and we cannot exclude a worsening additive effect of hypercapnia. Moreover, abnormal mechanical strains applied on the lung parenchyma during increased respiratory efforts associated with OSA were not taken into consideration in the present study. OSA has been reported in prevalent and incident case of IPF [8], and we tried to address the question whether OSA could modulate the course of pulmonary fibrosis. We showed that the major impact of IH on lung fibrosis was observed when IH started 14 days before induction of lung fibrosis by intratracheal instillation of BLM. The major pathogenic feature reported in IH and in related-comorbidities of OSA are oxidative stress and inflammatory response. However, BLM also induced an oxidative stress and inflammation [44]. In our study, we showed that the additive and worsening effect of IH on lung fibrosis involved the induction of pro-fibrotic markers and the expression of ER stress transcription factors. Interestingly, distinct UPR signalling pathways were activated by IH and BLM exposure. Unfortunately, the relationship between induction of ER stress and oxidative stress could not be investigated in our experimental model since BLM alone induces DNA strand breaks and localized oxidative stress. However, in our study we could not exclude a synergic effect of IH and BLM on lung fibrosis.

## 5. Conclusions

To conclude, this experimental study highlighted the critical role of intermittent hypoxia in the induction of lung Endoplasmic Reticulum (ER) stress in the context of lung fibrosis. Because lung ER stress plays a key role in the development of Idiopathic Pulmonary Fibrosis (IPF), our results support the concept that intermittent hypoxia related to Obstructive Sleep Apnea (OSA) should be considered as a potential worsening factor in IPF. Therefore, preventing deleterious effects of intermittent hypoxia with Continuous Positive Airwaiy Pressure (CPAP) could represent an attractive approach to limit IPF exacerbation. Interestingly, a recent study has demonstrated a positive effect of OSA treatment with CPAP on ER stress on obese subjects [45]. Therefore, it would be highly useful to investigate the potential impacts of CPAP on ER stress/pro-fibrotic marker expression in IPF patients and on their lung function evolution. 

## Figures and Tables

**Table 1:** Summary table of primers used for Real-Time Polymerase Chain Reaction.

Gene	Forward Primer (5′-3′)	Reverse Primer (5′-3′)
*Collagen 1a1*	GTGGTGACAAGGGTGAGACA	GAGAACCAGGAGAACCAGGA
*Collagen 3a1*	TACACCTGCTCCTGTGCTTC	CATTCCTCCCACTCCAGACT
*Serpin 1*	GCACAACCCGACAGAGACAA	ATGAAGGCGTCTCTTCCCAC
*Cxcl 12*	CCTTCAGATTGTTGCAAGGCTG	TCCTTTGGGCTGTTGTGCTT
*Pdgf β*	TCGCCTGCAAGTGTGAGACA	CCGAATGGTCACCCGAGCTT
*18S*	GTA AGT GCG GGC CAT AAG CTT	AGT CAA GTT CGA CCG TCT TCT CA

**Table 2:** Summary table of antibodies used for western blot analysis.

Antibodies (anti-)	Type	Host	Dilution	Reference	Manufacturer
β-ACTIN	Primary antibodies	Mouse	1/5000	A5316	Sigma
ATF6-N		Mouse	1/1000	Clone 1-7 MAB 6762	Abnova
ATF4		Rabbit	1/1000	SC-200 (C-20)	Santa Cruz
GRP 78		Rabbit	1/1000	ADI-SPA-7680050	Enzo
GRP 75		Rabbit	1/1000	D 175 9661L	Cell Signaling
sXBP1		Mouse	1/1000	SC-8015 (F-4)	Santa Cruz
Anti-mouse IgG HRP	Secondary antibodies	Goat	1/3000	P0447	Dako
Anti-rabbit IgG HRP		Goat	1/3000	P0448	Dako
